# Towards a comprehensive phylogeny of the large temperate genus *Pedicularis* (Orobanchaceae), with an emphasis on species from the Himalaya-Hengduan Mountains

**DOI:** 10.1186/s12870-015-0547-9

**Published:** 2015-07-11

**Authors:** Wen-Bin Yu, Min-Lu Liu, Hong Wang, Robert R. Mill, Richard H. Ree, Jun-Bo Yang, De-Zhu Li

**Affiliations:** Key Laboratory for Plant Diversity and Biogeography of East Asia, Kunming Institute of Botany, Chinese Academy of Sciences, Kunming, 650201 PR China; Center for Integrative Conservation, Xishuangbanna Tropical Botanical Garden, Chinese Academy of Sciences, Mengla, 666303 PR China; Plant Germplasm and Genomics Center, Germplasm Bank of Wild Species, Kunming Institute of Botany, Chinese Academy of Sciences, Kunming, 650201 PR China; Kunming College of Life Sciences, University of Chinese Academy of Sciences, Kunming, 650201 PR China; Royal Botanic Garden Edinburgh, 20A Inverleith Row, Edinburgh, EH3 5LR Scotland UK; Department of Botany, Field Museum of Natural History, Chicago, IL 60605 USA

**Keywords:** Adaptive radiation, Floral diversity, Phyllotaxy, Himalaya-Hengduan Mountains, Orobanchaceae, *Pediculari*s, Phylogenetic analysis

## Abstract

**Background:**

Striking interspecific variations in floral traits of the large temperate genus *Pedicularis* have given rise to controversies concerning infra-generic classifications. To date, phylogenetic relationships within the genus have not been well resolved. The main goal of this study is to construct a backbone phylogeny of *Pedicularis*, with extensive sampling of species from the Himalaya-Hengduan Mountains. Phylogenetic analyses included 257 species, representing all 13 informal groups and 104 out of 130 series in the classification system of Tsoong, using sequences of the nuclear ribosomal internal transcribed spacer (nrITS) and three plastid regions (*matK*, *rbcL* and *trnL-F*). Bayesian inference and maximum likelihood methods were applied in separate and combined analyses of these datasets.

**Results:**

Thirteen major clades are resolved with strong support, although the backbone of the tree is poorly resolved. There is little consensus between the phylogenetic tree and Tsoong’s classification of *Pedicularis*. Only two of the 13 groups (15.4 %), and 19 of the 56 series (33.9 %) with more than one sampled species were found to be strictly monophyletic. Most opposite-/whorled-leaved species fall into a single clade, i.e. clade 1, while alternate leaves species occur in the remaining 12 clades. Excluding the widespread *P. verticillata* in clade 1, species from Europe and North America fall into clades 6–8.

**Conclusions:**

Our results suggest that combinations of morphological and geographic characters associated with strongly supported clades are needed to elucidate a comprehensive global phylogeny of *Pedicularis*. Alternate leaves are inferred to be plesiomorphic in *Pedicularis*, with multiple transitions to opposite/whorled phyllotaxy. Alternate-leaved species show high diversity in plant habit and floral forms. In the Himalaya-Hengduan Mountains, geographical barriers may have facilitated diversification of species with long corolla tubes, and the reproductive advantages of beakless galeas in opposite-/whorled-leaved species may boost speciation at high altitude.

**Electronic supplementary material:**

The online version of this article (doi:10.1186/s12870-015-0547-9) contains supplementary material, which is available to authorized users.

## Background

*Pedicularis* L. comprises approximately 600 species [1, 2], and as such is the largest genus of Orobanchaceae (Lamiales) [3, 4]. Species of *Pedicularis* are biennial or perennial hemiparasitic herbs, and are primarily distributed in mountain ranges throughout the North Temperate zone [5, 6]. More than 350 species are recorded in the Himalaya-Hengduan Mountains, with some 75 % of these species endemic [7–9]. In this genus, most species have relatively narrow distributions [2], while only a few species, such as *P. oederi* Vahl and *P. verticillata* L., have much broader ranges that extend across the Northern Hemisphere [9–12].

Traditionally, *Pedicularis* was placed in tribe Rhinantheae of the Scrophulariaceae [1, 13], but on the basis of molecular evidence this tribe has since been transferred to Orobanchaceae and found to be polyphyletic [4, 14–17]. Phylogenetic analyses show that *Pedicularis* and *Phtheirospermum japonicum* (Thunb.) Kanitz, together with several New World hemiparasitic genera, are excluded from tribe Rhinantheae, and form a clade that corresponds to an amended tribe Pedicularideae [3, 14, 16, 18]. Hong [19] speculated that *Pterygiella* Oliver and *Xizangia* D.Y. Hong, two genera of the traditional tribe Rhinantheae endemic to China, shared morphological similarities with *Pedicularis* and *Phtheirospermum* Bunge ex Fischer & C.A. Meyer. However, recent molecular phylogenetic studies show that *Pterygiella* is close to tribe Rhinantheae [16], and furthermore, *Pterygiella* + *Xizangia* is supported as monophyletic with *Pseudobartsia* D.Y. Hong and other three species of *Phtheirospermum* [20]. *Pedicularis* is resolved as the sister to the rest of Pedicularideae [16].

The morphological characters that have received most attention in the taxonomic literature on *Pedicularis* are of corolla shape, leaf arrangement, and inflorescence structure. Floral traits that exhibit greatest interspecific variation are the shape of the corolla upper lip (galea) and the length of the corolla tube. In this context, four general corolla types have been recognized [6, 21, 22]: (i) short tubular corolla (<25 mm, hereafter) with beakless and toothless galea, (ii) short tubular corolla with toothed galea, (iii) short tubular corolla with beaked galea, and (iv) long tubular corolla (≥25 mm, hereafter) with beaked galea. It is noteworthy that the fourth type, i.e. the long tubular corolla bearing beaked galea, is restricted to the Himalaya-Hengduan Mountains. Li [2] hypothesized that floral evolution in *Pedicularis* may start as beakless and toothless, and subsequently be transformed to toothed and/or to beaked, culminating in the long tubular type. Analysis of the evolutionary histories of floral characters shows that the long tubular corolla appears to be derived from the short tubular corolla, toothed galea from toothless galea, while beaked galea arises first in a sub-basal clade corresponding to *P.* section *Lasioglossa*, then it has been lost several times in subsequent clades [21]. Phyllotaxy may be alternate, opposite, or whorled, and flowers may be borne on terminal inflorescences or solitary in the leaf axils. Tsoong [23] proposed that the opposite/whorled phyllotaxy should be derived from the alternate leaved phyllotaxy, rather than the reverse as suggested by Li [2].

Several classification systems have been proposed for *Pedicularis* [23] that differ notably in their emphasis on floral versus vegetative characters in recognizing infrageneric groups. The system of Maximowicz [24] divided *Pedicularis* into five “*tribes*” (misplaced taxonomic rank in accordance with the Melbourne Code of ICN [25]), four of them based on corolla types and one (*Verticillatae*) based on phyllotaxy. The subsequent systems of Prain [26], Bonati [27] and Limpricht [28] disintegrated *Verticillatae* and used floral characters exclusively to delimit primary infrageneric groups, and phyllotaxy to delimit lower taxa (subgroups, sections, or series). The system of Li [2, 29], which focused on the revision of the 282 species of *Pedicularis* then known from China, used vegetative characters to delimit three primary ‘*greges*’ (= groups [30], an informal taxonomic rank [25]). These were: group *Cyclophyllum*, with opposite/whorled leaves and terminal inflorescences; group *Allophyllum*, with alternate leaves, erect stems and terminal inflorescences; and group *Poecilophyllum*, with alternate or subopposite leaves and slender and diffuse or short stems and axillary or scattered inflorescences. Within these three groups, the species were classified hierarchically into sections and series primarily using vegetative and floral characters, respectively. This classification structure reflected an explicit hypothesis that similar corolla forms had evolved independently in different sections [6]. Tsoong [23, 31, 32] concurred with this view and proposed a global system for *Pedicularis* comprising 13 ‘*greges*’ (5 groups alternate-leaved, 1 group subopposite-/alternate-leaved, and 7 groups opposite/whorled-leaved) and 130 series. The Chinese species were classified into 13 groups and 113 series [9, 33].

The molecular phylogeny of *Pedicularis* has been investigated by Yang and colleagues [34, 35], Ree and colleagues [21, 36], Tkach et al. [37], and Robart et al. [38]. These studies found informative phylogenetic variation in sequences of the nuclear ribosomal internal transcribed spacer (nrITS) and the plastid regions *trnT*-*trnF*, *matK*, and *rps16*. They also revealed a general pattern of low congruence between morphology-based classifications and molecular phylogenies: few infra-generic taxa were confirmed to be monophyletic, and strongly supported clades did not in general correspond to recognized infra-generic taxa. Recently, Tkach et al. [37] sampled 218 taxa to reconstruct the largest phylogeny of *Pedicularis* so far, in which eight major clades were resolved. The focus of their study was on the biogeographical origin and evolution of Arctic species, not infrageneric classifications. Furthermore, around 100 species from the biodiversity center, the Himalaya-Hengduan Mountains, were sampled. Because many endemic series in this region have not been sampled in previous studies, a global phylogeny of *Pedicularis* does not yet exist. In addition, an endemic informal group *Cyathophora* is resolved as monophyletic; however, potential gene flow between species was discovered [39, 40]. This phenomenon may reduce phylogenetic resolution or cause phylogenetic incongruence between different genes. Based on a community sampling in the Hengduan Mountains, however, Eaton et al. [36] revealed that divergence in floral characters effectively reduced pollen transfer among sympatrically and synchronously flowering species. In large-scale phylogenies of *Pedicularis*, therefore, incongruence between genes is found at a low level [21, 37].

In this study, our primary objective was to reconstruct a phylogeny of *Pedicularis* with greatly expanded taxon sampling from China, particularly in the Himalaya-Hengduan Mountains, in order to provide a more comprehensive framework for infrageneric classification of *Pedicularis*. Based on the phylogeny of *Pedicularis*, our further aims were to: (i) compare the resulting molecular topology with traditional classifications; and (ii) explore systematic implications of vegetative and floral characters. Sequences from both the nuclear and chloroplast genomes were used to address these questions.

## Methods

### Taxon sampling

A total of 337 accessions of *Pedicularis* representing 257 species were sampled, including 235 out of 365 species recorded in China (64.4 %). This represents all 13 informal groups and 104 out of 130, or 80 %, of the global number of series (99 out of 113, or 87.6 %, of the series represented in China), following the system of Tsoong [23, 31–33]. To make it comparable, in the following sections, the numbers in parentheses after group names of Tsoong’s system refer to the number assigned to the group as circumscribed in this paper and listed in Additional file 1. For the outgroups, 15 genera of Orobanchaceae were selected on the basis of previous studies [4, 14, 16]. Of these, four genera (*Melampyrum*, *Phtheirospermum*, *Pterygiella* and *Xizangia*) belong to the traditional tribe Rhinantheae [1, 13]. All taxa included in this study are listed in Additional file 1, with their group and series assignments, voucher information and GenBank accession numbers.

### DNA extraction and amplification

Total genomic DNA extraction and protocols for polymerase chain reaction (PCR) amplification and sequencing followed Yu et al. [41]. One nuclear gene (nrITS) and three chloroplast gene/regions (*matK*, *rbcL*, and *trnL-F*) were sequenced in this study (Table 2). Primer information of nrITS, *matK* and *rbcL* was presented by Yu et al. [41]. The plastid *trnL-F* region (including *trnL* (UAA) intron and *trnL-F* intergenic spacer) was amplified and sequenced using the primers ‘c’ and ‘f’ , and some samples were sequenced additionally with an internal primer ‘d’ for the 5′ end [42].

### Phylogenetic analysis

All newly obtained raw sequences were assembled and edited using Geneious version 7.0 [43]. Boundaries of DNA regions were verified by BLAST searches in GenBank. Preliminary alignments were produced using MAFFT version 6.0 [44], then adjusted manually in BioEdit version 7.0 [45]. Aligned matrices of four DNA regions were combined using SequenceMatrix version 1.7 [46]. The nrITS and concatenated plastid datasets were analyzed separately and in combination. No nucleotide positions were excluded from the analyses.

Bayesian Inference (BI) and Maximum Likelihood (ML) methods were used for phylogenetic reconstruction. Partioned BI analyses were performed using MrBayes version 3.2.3 [47], with DNA substitution models selected for each gene partition by the Bayesian information criterion (BIC) using jModeltest version 2 [48, 49]. The best-fit models used for each DNA region are listed in Table 1. Markov Chain Monte Carlo (MCMC) analyses were run in MrBayes for 10,000,000 generations for each dataset, with each run comprising four incrementally heated chains. The BI analyses were started with a random tree and sampled every 1000 generations. The first 20 % of the trees were discarded as burn-in, and the remaining trees were used to generate a majority-rule consensus tree. Posterior probability values (PP) ≥ 0.95 were considered as well supported [50–52]. ML tree searches and bootstrap estimation of clade support were conducted with RAxML [53]. These analyses used the GTR substitution model with gamma-distributed rate heterogeneity among sites and the proportion of invariable sites estimated from the data. For the ML analyses, bootstrap support (BS) ≥ 70 % were considered well supported [54]. Both BI and ML analyses, as well as jModeltest, were performed at the CIPRES Science Gateway (http://www.phylo.org).

**Table 1:** Basic information for sequence characters and best-fit model of Bayesian information criterion (BIC) for Bayesian inference

Parameters	nrITS	Chloroplast genes				Total
		*matK*	*rbcL*	*trnL-F*	Concatenated dataset	
No. of accessions (*Pedicularis*)	341 (326)	327 (314)	321 (309)	322 (307)	343 (328)	352 (337)
Aligned length (bp)	680	777	654	1577	2942	3622
Variable sites/Parsimony informative sites						
*Pedicularis* + outgroups	459/377	427/394	167/99	702/433	1296/836	1755/1213
*Pedicularis*	428/336	377/267	133/87	619/395	1129/749	1577/1085
BIC model	GTR + I + G	TVM + G	TPM1uf + I + G	TPM1uf + G	--	--
-lnL	22752.7454	10062.8110	3971.1126	14883.0839	--	--
*K*	690	662	649	648	--	--
Frequency A	0.2147	0.3710	0.2661	0.3534	--	--
Frequency C	0.2449	0.1707	0.2195	0.1775	--	--
Frequency G	0.2362	0.1553	0.2082	0.1546	--	--
A↔C	0.9173	1.1879	1.0000	1.0000	--	--
A↔G	1.6908	2.5747	3.4921	1.8897	--	--
A↔T	1.3062	0.1754	0.4321	0.4951	--	--
C↔G	0.3371	0.7679	0.4321	0.4951	--	--
C↔T	2.8586	2.5747	3.4921	1.8897	--	--
G↔T	1.0000	1.0000	1.0000	1.0000	--	--
Gamma distribution shape parameter of variable sites	0.9960	0.7910	0.7000	0.7790	--	--
Proportion of invariable sites	0.2540	0.0000	0.5790	0.0000	--	--

Phylogenetic incongruence between BI and ML analyses using the same dataset was visually compared using TreeGraph version 2 [55]. The incongruence length difference test (ILD) [56] was not used to assess topological conflict between the nuclear and concatenated plastid datasets, because this analysis has been shown to be misleading [37, 57]. We used a conservative PP ≥ 0.95 and BS ≥ 70 % as threshold for identifying significantly incongruent clades. Due to only a weak conflict, we combined the total datasets for the additional phylogenetic analyses.

### Reconstruction of ancestral states of morphological characters

Ancestral states of four morphological characters (phyllotaxy, corolla tube, galea tooth, and galea beak) were inferred by mapping characters on to the BI tree of the total dataset. Outgroups were removed. Parsimony reconstructions were performed using Mesquite version 2.75 [58]. Following Ree [21], each character was coded categorically with two states, 0 and 1. States were assigned as follows: phyllotaxy, alternate/sub-opposite (0) or opposite/whorled (1); corolla tube, short (0) or long (1); and galea teeth, absent (0) or present (1); galea beak, absent (0) or present (1).

## Results

### Nuclear, plastid, and concatenated datasets

Sequence characteristics of four DNA regions, and the concatenated plastid and total datasets are summarized in Table 1. The numbers of both variable sites and parsimony informative sites were highest for nrITS, followed by *trnL-F*, *matK* and *rbcL*. The *matK* alignment was shorter than that of *trnL-F*, but the proportions of both variable sites and parsimony informative sites of *matK* (48.5 % and 34.4 % respectively) were higher than those of *trnL-F* (39.2 % and 25.9 % respectively) in the matrix excluding outgroups. The best-fit BIC models for four DNA regions were independent, thus the BI analyses of the concatenated plastid and total datasets were partitioned using a specific model for each DNA region.

**Fig. 1:**
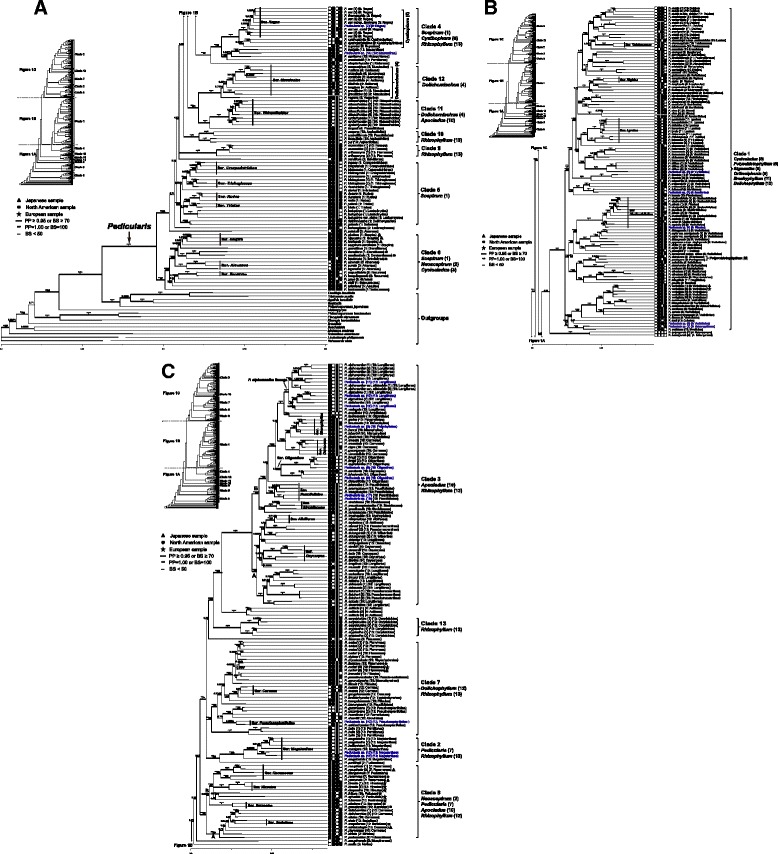
Phylogeny of *Pedicularis* inferred from Bayesian Inference (BI), Maximum Likelihood (ML) methods using nuclear ribosomal internal transcribed spacer (nrITS) and three plastid *(matK*, *rbcL* and *trnL-F*) datasets. Topology shows the majority rule consensus of the Bayesian inference tree using the combined nrITS and plastid datasets. Values above the branches indicate BI posterior probability (PP) ≥0.50 and ML bootstrap support (BS) ≥50. Infra-generic group number and series names are listed in parentheses after each species name in accordance with the system of Tsoong [23, 31–33]. Infra-generic informal group name and number (in parentheses) are summarized under the major clade number. Samples from the Europe, North America and Japan, are annotated by the pentagram (★), circle (●) and triangle (▲), respectively. Morphological character states of *Pedicularis* are represented by the grid of boxes to the right, where columns correspond to phyllotaxy, corolla tube, galea tooth, and galea beak, respectively; filled boxes (■) indicate spiral phyllotaxy, short corolla tubes, toothless galeas, beakless galeas, while unfilled boxes (□) indicate opposite/whorled phyllotaxy, elongate corolla tubes, toothed galeas, and beaked galeas

Topology was consistent between BI and ML trees. Both BI and ML analyses strongly supported the genus *Pedicularis* as monophyletic (total dataset: Fig. 1, nrITS dataset: Additional file 2, the plastid dataset: Additional file 3). However, in all analyses the resolution of backbone branches was generally poorly supported or unresolved. Plastid and total datasets recovered 13 well supported clades that merit comment (Table 2). In addition, phylogenetic placements of eight species (*P. petelotii*, *P. lachnoglossa*, *P. salviiflora*, *P. batangensis*, *P. mollis*, *P. moupinensis*, *P. flexuosa*, and *P. axillaris*) were not resolved, and they tended to be treated as separated clades or lineages (Fig. 1). Eight clades have been reported in previous studies [21, 37], so previous clade numbering (as clades 1–8) was followed. Five new clades resolved in this study are consequently here labeled clades 9–13. These clades received congruent support in separate analyses using the nrITS and plastid datasets, with the exception of clades 6, and 8, which were not supported by nrITS dataset (Table 2, Additional file 2). In general, clade resolution and support values were higher in trees from the total dataset analyses compared with separate analyses of the nrITS and plastid datasets.

**Table 2:** Summary for support values of major clades on the basis of Bayesian inference (BI) and Maximum likelihood (ML) methods

Major clades in *Pedicularis*	Phylogenetic analyses (BI/ML)		
	nrITS	Concatenated plastid dataset	Total
Clade 1	0.99/69	1.00/92	1.00/100
Subclade 1A	--	1.00/80	1.00/62
Subclade 1B	--	--	1.00/67
Subclade 1C	--	1.00/89	1.00/79
Clade 2	0.89/42	1.00/91	1.00/85
Clade 3	0.98/96	1.00/100	1.00/100
Subclade 3A	--	--	1.00/85
Subclade 3B	--	--	1.00/88
Clade 4	1.00/80	1.00/100	1.00/81
Clade 5	0.94/45	0.82/38	1.00/86
Clade 6	--	1.00/79	1.00/91
Clade 7	0.84/46	0.87/72	1.00/88
Clade 8	--	1.00/42	1.00/92
Subclade 8A	0.89/44	−−/−−	0.95/62
Subclade 8B	1.00/57	0.52/62	1.00/98
^a^Clade 9	1.00/100	1.00/100	1.00/100
^a^Clade 10	1.00/99	1.00/99	1.00/100
^a^Clade 11	0.99/97	0.93/87	1.00/100
^a^Clade 12	1.00/78	1.00/99	1.00/100
^a^Clade 13	1.00/100	1.00/100	1.00/100

^a^Newly-resolved clade

### Phylogenetic analyses

The phylogenetic tree using the total data is given in Fig. 1. There was little consensus between this phylogeny and the taxonomic hierarchy of Tsoong’s system (Additional file 1). Only two of the 13 informal groups (*Polyschistophyllum* and *Cyathophora*: 15.4 %) and 19 (33.9 %) of the 56 series with more than one sampled species (series *Carnosae*, *Cernuae*, *Corydaloides*, *Hirsutae*, *Macranthae*, *Megalanthae*, *Pseudoasplenifoliae*, *Recurvae*, *Reges*, *Rhinanthoides, Rigidae*, *Rudes*, *Sceptra*, *Strobilaceae*, *Surrectae*, *Sudeticae*, *Tatsienenses Trichoglossae*, and *Tristes*) were found to be strictly monophyletic. In addition, series *Aloenses*, *Asplenifoliae*, *Cheilanthifoliae*, *Craspedotrichae*, *Lyratae*, *Microphyllae*, *Muscicolae*, *Oliganthae*, *Oxycarpae*, *Paucifoliatae*, and *Racemosae* were paraphyletic, but in each case forming a clade that included at least two-thirds of species from the same series.

Thirteen major clades, labeled 1–13, are addressed below in ascending order as they appear in the tree (Fig. 1). Support values are specified in the following text as BI PP/ML BS, e.g. 1.00/92 (PP/BS value). Phylogenies of the plastid (1.00/92) and total datasets (1.00/100) strongly supported clade 6 as the sister clade to the rest of *Pedicularis* (1.00/99). However, clade 6 was resolved as paraphyletic in the nrITS tree (Additional file 2). Within clade 6, 14 alternate-leaved species were from the informal groups *Sceptrum* (1) and *Neosceptrum* (2) in Tsoong’s system, with only three species of series *Aloenses* from the informal group *Cyclocladus* (3) with opposite leaves. Series *Aloenses* (excluding *P. petelotii*, 1.00/100), *Recurvae* (1.00/100), and *Sceptra* (0.68/51) in clade 6 were more or less supported as monophyletic.

Two species, *P. petelotii* (series *Aloenses*) and *P. lachnoglossa* (series *Lachnoglossae*, one species in Himalaya), formed a grade, followed by the alternate-leaved clade 5. Clade 5 was strongly supported by the total datasets (1.00/86), but it was weakly supported using the nrITS and plastid datasets (Additional files 2, 3 and Table 2). This clade contains 16 species representing six series of the informal group *Sceptrum* (1) in Tsoong’s system. The monophyly of series *Craspedotrichae* (1.00/92), *Rudes* (1.00/100), *Trichoglossae* (1.00/94), and *Tristes* (1.00/95) was well supported.

The relationship between *P. salviiflora* (series *Salviiflorae*, one species in SW China) and clade 9 was not resolved. Clade 9 contained two species, *P. muscoides* (series *Roseae*) and *P. orthocoryne* (series *Flammeae*). Furthermore, all three datasets strongly supported this clade as monophyletic (Table 2). This phenomenon was also found in the newly resolved clade 10, which was strongly supported as the sister to the remaining eight clades in the BI analysis (PP = 1.00, Fig. 1). It included three species from series *Asplenifoliae*, as well as *P. umbelliformis* (series *Paucifoliatae*). The phylogenetic relationship of clade 10 was incongruent between nrITS and the plastid trees. The nrITS dataset supported clade 10 as the sister of clade 12 (1.00/78, see Additional file 2), but the plastid dataset did not resolve its position (Additional file 3).

Clades 11 and 12 were strongly supported as the sister to the remaining seven clades in the BI analysis (PP = 0.96, Fig. 1a). Clades 11 and 12 were strongly supported as sister groups by the plastid (1.00/98) and total datasets (1.00/96). However, the nrITS dataset weakly supported clade 11 close to clade 4 (PP = 0.82, see Additional file 3), and strongly supported clade 12 as sister to clade 10 (see above). Thus, clades 11 and 12 were treated as two independent clades in this study. Within clade 11, series *Rhinanthoides* (1.00/100) was strongly supported as monophyletic, and the relationship between *P. cyclorhyncha* and *P. rhinanthoides* was well resolved using the total dataset. Clade 12 corresponded to the informal group *Dolichomischus* in Tsoong’s system, including representatives of series *Axillares* (but excluding the type species *P. axillaris*, which is the sister of clade 3), *Omiianae* and *Muscicolae*, although none of these series were recovered as monophyletic.

Clades 1 and 4, and *P. batangensis* (series *Batangenses*, one species in SW China) formed a clade that was supported by the plastid (1.00/72, Additional file 3) and total datasets (PP = 0.88, Fig. 1). The nrITS (PP = 0.51) and total datasets (PP = 0.63) weakly supported *P. batangensis* close to clade 1, while the plastid dataset strongly supported *P. batangensis* close to clade 4 (1.00/97). Clade 4 contains all species of the informal group *Cyathophora* and five alternate-leaved species (Fig. 1a). *Pedicularis streptorhyncha* (series *Kialenses*) is placed at the base of this clade, and the remaining four alternate-leaved species were the sister subclade of the monophyletic informal group *Cyathophora*. Within the informal group *Cyathophora*, series *Reges* and another three species with large purple corollas formed two well-supported subclades, respectively.

Clade 1 contains 82 species, representing six opposite-/whorled-leaved informal groups and 27 series (Additional file 1, Fig. 1b). Informal group *Polyschistophyllum* and series *Rigidae* were both monophyletic and nested within the clade. The clade did not, however, include any of the following: the whorled-leaved informal group *Cyathophora* (in clade 4); series *Molles* (*P. mollis*) and *Moupinenses* (*P. moupinensis*) (both basal to clade 8); series *Tantalorhynchae* (*P. tantalorhyncha* in clade 7); the opposite-leaved series *Aloenses* (three species in clade 6, and the isolated *P. petelotii*), *Atrovirides* (*P. sherriffii* in clade 7), *Cernuae* (*P. cernua* and *P. gongshanensis* in clade 7), *Pseudoasplenifoliae* (three species in clade 7), *Remotilobae* (*P. remotiloba* in clade 7), and *Salviiflorae* (*P. salviiflora*, located between clades 5 and 9); and the isolated *P. flexuosa* (series *Flexuosae*: located between clades 7 and 13). In terms of distribution, only *P. chamissonis* from Japan, and two samples of *P. verticillata* from Japan and Europe, were from outside China or the Himalayas; the remaining 108 samples representing 81 species (including three samples of *P. verticillata*) were from China, extending to Himalayas, particularly in southwestern (SW) China. Within clade 1, we recognize three subclades, labeled A-C in Fig. 1b. Subclade 1A consisted of 12 species (16 samples), 8 of those from series *Verticillatae* and only *P. smithiana* with beaked galea. Within the subclade of five samples representing the circumboreal species *P. verticillata*, the samples from Europe and Japan grouped together, while the samples from China tended to be distantly related.

Subclade 1B (1.00/67) contained 20 species (30 samples), with monophyletic informal group *Polyschistophyllum* nested within it. Series *Cheilanthifoliae* (three species) and *Plicatae* (two species), and *P. rupicola* formed a complex lineage. Four species with multiple samples did not resolve as monophyletic. In addition, *P. roylei* var. *brevigaleata* was close to *P. likiangensis*, not to other samples of *P. roylei*.

Subclade 1C (1.00/62) included 50 species (65 samples), with two long-tubed species, i.e. *P. urceolata* (series *Urceolatae*) and *P. oxyrhyncha* (series *Flexuosae*). The monophyletic series *Rigidae* and *Tatsienenses*, and paraphyletic series *Lyratae* were included in this subclade. It is noteworthy that *Pedicularis debilis*, with six sampled individuals, tended to be resolved as at least three species. In addition, *P. kansuensis* (series *Verticillatae*) and *P. lutescens* (series *Lyratae*) were paraphyletic.

*Pedicularis mollis* (series *Molles*, one species in Himalaya) and *P. moupinensis* (series *Moupinenses*, one species in SW China) loosely formed a grade (PP = 0.72) underlying a superclade consisting of clades 2, 3, 7, 8, and 13, plus *P. flexuosa* (series *Flexuosae*) and *P. axillaris* (PP = 0.93). This latter superclade was strongly supported clade by BI analysis (PP = 1.00), while the clade (clades 13 + *P. axillaris* + clade 3) was strongly supported by the total (1.00/70) and plastid (1.00/89) datasets. *Pedicularis axillaris* was supported as the sister of clade 3 (total dataset: 1.00/82; nrITS dataset: PP = 0.72; plastid dataset: 0.74/72), while this species was excluded from clade 3 by previous treatments [21, 37].

Clade 8 was strongly supported only by the total dataset (1.00/92) and the plastid dataset using BI analysis (PP = 1.00). The nrITS dataset resolved clade 8 (PP = 0.86) as paraphyletic, including clade 13 that close to subclade 8A (PP = 0.52) (Additional file 2). Clade 8 contained 19 species (22 samples) belonging to ten series and four informal groups (Fig. 1c). Two subclades could be recognized, labeled 8A (8 species) and 8B (11 species: Fig. 1c). Collectively, the geographic range of clade 8 extends across Asia (including Japan), Europe, and North America, whereas none of its species occur in the Himalaya-Hengduan Mountains. The monophyly of series *Hirsutae*, *Surrectae* (both in subclade 8B) and series *Sudeticae* (subclade 8A) was supported.

Clade 2 was resolved by all analyses (Table 2). *Pedicularis pantlingii* (series *Furfuraceae*) was resolved at the base of this clade. The remaining species corresponded to series *Megalanthae*, endemic to the Himalaya region. Morphologically, two samples from Xizang were identified in series *Megalanthae*, while they were different from two inland Chinese species *P. megalantha* and *P. megalochila*.

Three samples of *P. bella* (series *Pumiliones*) were monophyletic at the base of clade 7. This clade contained 19 species (28 samples) from two informal groups presently classified in 12 series: eleven alternate-leaved species represent seven series of the informal group *Rhizophyllum*, and eight opposite-/whorled-leaved species represent five series of the informal group *Dolichophyllum*. The six samples representing *P. oederi* (series *Flammeae*) are not monophyletic: the Japanese and European samples formed a moderately supported clade (0.89/67) that also included *P. flammea*, whereas the Chinese samples were clustered with *P. rhynchodonta* and *P. stylosa* (0.98/61). Monophyly of series *Cernuae* was weakly resolved (PP = 0.64).

Clade 13 corresponded to series *Corydaloides*, including two species endemic to the Himalaya-Hengduan Mountains. As with clades 9–12, this clade was strongly supported by all analyses (Table 2). Monophyly of the two species was well resolved.

Clade 3 contained 59 species (71 samples) from the informal groups *Apocladus* (10) and *Rhizophyllum* (13) with alternate leaves, representing 15 series. This clade was divided into two well-supported subclades, labeled 3A and 3B (Fig. 1c). Subclade 3A included 22 species (27 samples). Species in the clade *P. cranolopha – P. decorissima* (1.00/100) belonged to series *Longiflorae* except for *P. fletcheri*. In addition, except for *P. macilenta* (in subclade 3A, clustering with five species of series *Oliganthae*), the remaining five species of series *Oxycarpae* clustered together (0.67/72). Series *Albiflorae* was paraphyletic, including *P. mychophila* (series *Mychophilae*).

Subclade 3B (1.00/85) contained 37 species (44 samples), of which only two species, *P. henryi* and *P. tenuisecta*, were beakless. In this subclade, long tubular species from series *Longiflorae* (1.00/100) formed a strongly supported group here termed the *P. siphonantha* lineage (Fig. 1c). This group corresponded to species in series *Longiflorae* with purple/red/pink corollas with twisted beaks. In addition, eight beaked species (from series *Amplitubae* and *Oliganthae*) with a reflexed tooth on either side of the lower margin were strongly supported to be sister to each other, and they were sister to the lineage within this subclade. Series *Carnosae* (1.00/100) and *Strobilaceae* (1.00/100) were strictly monophyletic, and series *Microphyllae*, *Oliganthae* and *Paucifoliatae* were paraphyletic.

### Reconstruction of ancestral states of morphological characters

**Fig. 2:**
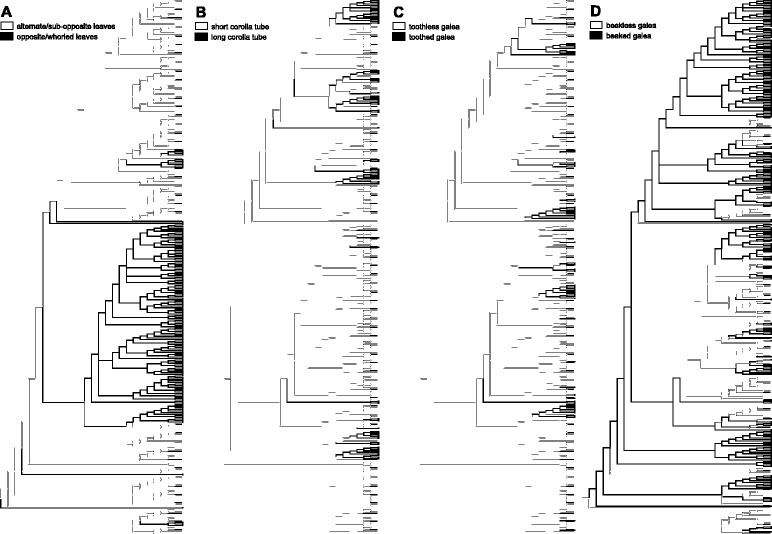
Evolutionary history of four morphological characters, phyllotaxy (**a**), corolla tube (**b**), galea tooth (**c**), and galea beak (**d**)

Evolutionary patterns of four morphological characters are shown in Fig. 2. Our results showed that alternate/sub-opposite leaves had been transformed to opposite/whorled leaves at least nine times (Fig. 2a), long-tubed corollas were derived from short-tubed corollas at least 21 times (Fig. 2b), toothed galea from toothless galea at least 26 times (Fig. 2c), and beaked galea had been gained three times in the basal clade, then it was lost more than 20 times; independent losses were particularly numerous in clade 1 (Fig. 2d).

## Discussion

### Comparisons between molecular phylogenies and morphological classifications

To date, this is the largest phylogeny assembled for *Pedicularis*, representing all 13 informal groups, 80 % of the total series and one-third of the total species [23, 31–33]. Overall, the separated nrITS and the plastid datasets, and the total dataset are informative in resolving relationships among recently diverged nodes, but provide poor resolution and support for earlier divergences. Short branch lengths for the stem of 13 well supported clades, and long branch lengths for some tip branches/nodes, imply that the most recent common ancestor (MRCA) of *Pedicularis* may have undergone rapid radiations at the early evolutionary stages.

It is not rare that phylogenetic topologies are incongruent with traditional classifications, because classical taxonomy sometimes emphasized homoplastic characters as the key criteria to arrange the system. In *Pedicularis*, floral characters were emphasized in several classifications [24, 26–28] before Li [2, 29]; however, floral characters show a higher degree of homoplasy than that of phyllotaxy [2, 6, 21, 29]. The current study, as well as that of Ree [21], both support the hypothesis that classifications are relatively stable on the basis of phyllotaxy. Eleven of the 13 clades are alike in vegetative characters, e.g., clade 1 is composed of opposite-/whorled-leaved species, clades 2, 3, 5, and 8–13 share alternate leaves, and clade 7 has membranous scales at the base of the stems. Clade 4 comprises both whorled-leaved and alternate-leaved species, and monophyly of the whorled-leaved informal group *Cyathophora* is strongly supported. One exception for the floral type is clade 6 which is characterized by having a “Capitata-type” corolla (i.e. corolla tube straight, lower lip apposed or erect [23]).

Our results agree to some extent with the principles underlying Tsoong’s system of infrageneric classification, particularly as to phyllotaxy [23, 31, 32]. In Tsoong’s system, the informal groups *Sceptrum* (1), *Neosceptrum* (2), *Pedicularis* (7), *Apocladus* (10) and *Rhizophyllum* (13) are alternate-leaved, while informal group *Dolichomischus* (4) is opposite-, subopposite- or alternate-leaved with slender and diffuse stems, and informal groups *Cyclocladus* (3), *Polyschistophyllum* (5), *Cyathophora* (6), *Sigmantha* (8), *Orthosiphonia* (9), *Brachyphyllum* (11) and *Dolichophyllum* (12) are opposite-/whorled-leaved. Among the 13 informal groups, groups *Polyschistophyllum* (5) and *Cyathophora* (6), both of which were thoroughly sampled by us, were strongly supported as monophyletic (Additional file 1). Species of informal group *Cyathophora* (6) are whorled-leaved, while all five sister species are alternate-leaved. All species of informal group *Polyschistophyllum* (5) and almost all species of the other five opposite-/whorled-leaved informal groups fall into clade 1. Nine alternate-leaved species of informal group *Dolichomischus* (4) fall into clade 12, whereas of three excluded species, the strictly opposite-leaved *P. batangensis* is close to clade 1, the subopposite-leaved *P. axillaris* is close to clade 3, and the alternate-leaved *P. fargesii* falls in clade 11. However, because of the fact that species of the five other alternate-leaved informal groups fall into 12 of the 13 clades (i.e. all except clade 1), the evolutionary significance of phyllotaxy should be treated with caution.

As to the other informal groups, species of group *Sceptrum* (1) are clustered in clades 5 and 6, except *P. streptorhyncha* of series *Kialenses* (in clade 4); *P. streptorhyncha* is the only sampled species with twisted corolla beak in informal group *Sceptrum* and the phylogenetic placement suggests that its taxonomic position may be incorrect. Three species of informal group *Neosceptrum* (2) fall into clade 6; of the other three species, the position of *P. lachnoglossa* is not resolved, while *P. striata* and *P. proboscidea* are located in clade 8. As for informal group *Pedicularis* (7), series *Carnosae*, *Microphyllae* and *Polyphyllatae* (all endemic to SW China) form a well-supported subclade in clade 3, while series *Pedicularis* and *Racemosae* (which occur throughout the North Temperate Zone) are clustered in clade 8. In addition, *P. pantlingii* of series *Furfuraceae* is nested as the base of clade 2.

The situation of the informal group *Apocladus* (10) resembles that of group *Pedicularis* (7). Five series endemic to SW China are included in clade 3, and five series distributed throughout the North Temperate Zone in clade 8. The two sampled species of series *Rhinanthoides* form clade 11, which also includes *P. fargesii* (series *Phaceliifoliae*). Species of the informal group *Rhizophyllum* (13) was highly polyphyletic, with sampled species being distributed in eight clades (3, 4, 7, 8–10, 12, and 13). Among these, clades 9, 10 and 13 contain species only from this group. Except for *P. umbelliformis*, all species of series *Franchetianae*, *Longiflorae*, *Mychophilae*, *Paucifoliatae* and *Pseudomacranthae* fall into the clade 3, with all species of series *Albiflorae* and *Pseudomacranthae* being included in subclade 3B. The sampled species of series *Filiculae* (excluding *P. tsangchanensis*), *Flammeae*, *Macrorhynchae*, *Pseudo-oederianae*, and *Rhynchodontae* form a strongly-supported clade 7. Series *Hirsutae* is monophyletic in clade 8. Interestingly, of the two species of series *Pumiliones*, *P. bella* falls into clade 7, but *P. przewalskii* is the sister of informal group *Cyathophora* in clade 4. This result strongly suggests that series *Pumiliones* is artificial and confirms the work of Ree [21] who also found that these two species were resolved in very different positions on the tree.

Based on the above comparisons of traditional classifications with molecular phylogenies, we reconfirm that vegetative traits are more reliable than floral traits in the infra-generic classification in *Pedicularis* [2, 21, 23]. Phyllotaxy is a key trait for understanding the classification of *Pedicularis*, while leaf morphology (e.g., petiole, blade and leaflets) and stem form are significant in the subdivisions of the classification. Some floral characters are conservative in the clades with mixed phyllotaxy, such as the “Capitata-type” corolla in clade 6 and the “Flammea-type” corolla (i.e. corolla tube abruptly bent forwards near apex, lower lip spreading at ± right angles to the tube and upper lip [23]) in clade 7. In addition, geographic distribution appears to carry some important phylogenetic signals. For example, most species of clades 6 and 8 are distributed throughout the Northern Temperate zone or are endemic to Japan or Europe and North America, whereas all species of clades 2, 4, 5, and 9–13 are endemic to SW China or the Himalaya-Hengduan Mountains. It seems that the Japanese/European/North American species have been derived from Himalayan ancestors [2, 6, 28, 38]. Nevertheless, molecular dating and biogeographic analyses should be applied to validate these hypotheses in the future.

### Systematic implications of vegetative characters

Whether the alternate or the opposite/whorled leaf arrangement is the ancestral trait in *Pedicularis* has been a matter for debate. Li [2] favored the view that the opposite/whorled status was primitive, while Tsoong [23] took the opposite viewpoint. Our results support the viewpoint of Tsoong [23] that alternate leaves could be plesiomorphic in *Pedicularis*, because the most basal clade 6 is mainly alternate-leaved (except series *Aloenses*). Alternate-leaved species of *Pedicularis* occur in 12 clades, indicating that alternate leaves may be a symplesiomorphic trait. Alternate leaves have independently evolved to opposite/whorled leaves at least nine times, such as in clades 6 (series *Aloenses*), 4 (informal group *Cyathophora*), 1, and 7 (series *Cernuae*). In addition, some stem characters are associated with alternate leaves. For example, membranous scales at the stem base occur in clades 7, 9 and 10, and slender and diffuse stems with axillary flowers are found in species of clade 12, *P. axillaris* (sister to clade 3) and *P. gruina* (in clade 3). Generally, species with slender, diffuse stems grow under moist forests or shrubs and have long corolla-tubes or long pedicels, which may serve to elevate flowers away from diffuse, leafy stems. The membranous scales represent the persistent petioles of the withered basal leaves, and this character may be a byproduct of the perennial habit and high-altitude/-latitude habitat of these species.

In *Pedicularis*, leaf morphology may also have significant implications for systematics. Characters of the petiole (leaf base) provide evidence for supporting the monophyly of clade 5 and the informal group *Cyathophora* in clade 4. Informal group *Cyathophora* is characterized by having perfoliate leaf bases that form a cup-like structure around the stem at each node. All classification systems have emphasized this special character for placing the species of this group as a cohesive unit, although it includes all four general corolla types of *Pedicularis* [2, 23]. Molecular phylogeny strongly supported the morphological basis of this group [21, 34, 40]. The cup-like bracts filling with rainwater may repel nectar robbers and/or seed herbivores [59].

Clade 5 is characterized by having clasping leaf bases, with pinnatifid leaves and pubescent corolla beaks. Short branch lengths of the clade suggest that clade 5 may have undergone a rapid radiation at high altitudes. Fleshy leaves rarely occur in *Pedicularis*, and this feature can be used to identify the lineage of *P. dichotoma* – *P. scolopax* in the subclade 1B and the lineage of series *Carnosae* in clade 3. The former lineage prefers dry and warm riverside habitats, and the latter lineage mainly occurs in limestone-associated meadows. *Pedicularis integrifolia* in subclade 1B also has fleshy leaves, and it is widely distributed in dry alpine meadows. Fleshy leaves may thus be a trait of functional importance in dry habitats, and it has been gained at least twice in clades 1 and 3.

### Floral diversification and radiation in the Himalaya-Hengduan Mountains

Corolla tube length and galea structure show striking interspecific variations in *Pedicularis*. Long-tubed corollas appear to have been derived from short-tubed corollas several times, while the directionality of change in galea structure is less certain [21]. Short-tubed species can bear all three types of galea (beakless and toothless, beakless and toothed, and beaked), while all long-tubed species are beaked (only *P. cyathophylloides* has an obscure beak). Studies of development of the upper lip show that different expansion patterns of the two upper petals determine whether the galea will or not develop a beak or teeth [60]. The evolution of galea teeth shows high levels of homoplasy [21], but galea teeth are synapomorphic for the monophyletic series *Lyratae*, *Rigidae* and *Reges*. Developmental patterns of the two major teeth are consistent across three toothed species, *P. comptoniifolia*, *P. lutescens* and *P. rex* [60], while the unsealed petal tips of *P. lutescens* (series *Lyratae*) have jagged or spinose margins on both inner sides that develop into pairs of small teeth. Two major teeth resulted from the expansion and elongation of the separated tips of the two upper petals. Initiation of two teeth occurs in the beaked species *P. cephalantha* (series *Oliganthae*) during the development of the beak [60] showing that the beak and tooth can simultaneously develop within the same flower. In the traditional classifications [29, 33], species of series *Amplitubae* and *Oliganthae* have beaked corollas with a pair of teeth at the margin of the galea. However, these species fell into two lineages in subclade 3A, indicating that the evolution of galea teeth in the species with a beaked galea may be similar to that of species with beakless galea.

In *Pedicularis*, the galea beak is the result of elongation of the two fused upper petals [60]. Transitions from beakless to beaked galeas appear to be associated with dramatic changes in nectar production and the foraging behavior of pollinators [21, 61, 62]. The nectarless beaked corollas force bumblebees to dislodge pollen through the funnel-shaped beak by vibrating their wings (buzz-pollination), so the beak can ensure pollination accuracy and efficiency [63–65]. Meanwhile, a pollen dispensing mechanism for the beaked corollas [66] enhances pollination success of the nectarless species of *Pedicularis* by increasing the visitation times [63, 67]. Increased pollination accuracy may reduce the probability of interspecific pollen deposition [36, 68, 69]. Specialization of the corolla beak may be associated with species diversity in *Pedicularis*, since beaked species account for 66 % of the 352 species in China [9] as well as 96.6 % of the species in clade 3. However, this hypothesis may not hold true in the case of clade 1, in which beakless species (toothless and toothed) account for 45 of the 82 (54.9 %) species in the clade. Why do opposite-/whorled-leaved species tend to have more beakless corollas? We suggest that beakless corollas are adapted to generalized pollination [61, 70]; this may be of great advantage in the opposite-/whorled-leaved species, because most such species are annual or biennial and monocarpic. Diverse pollinators [61, 71, 72] and autonomous selfing [73, 74] are only found in the beakless species, which may provide reproductive assurance when bumblebees are scarce at the high altitudes and latitudes where these species occur. In addition, a long and upright inflorescence with 2–4 flowers at each node enlarges floral displays for pollination attractiveness in the opposite-/whorled-leaved species. This may reduce reproductive resources to invest in development of corolla beak and tube, indicating a trade-off hypothesis between flower number and corolla size [75, 76]. Therefore, pollinators and/or reproductive adaptation put high selective pressure on evolution of corolla galea of *Pedicularis* in the Himalaya-Hengduan Mountains. Morphological diversification of corolla galea may reduce reproductive interference among co-occurring *Pedicularis* [36, 64], but also facilitate speciation in the recently derived group.

A total of 45 long-tubed species were sampled in this study. Of these, 22 species are in clade 3; six species in clades 2 and 12; three species in clade 4; and two species in clades 1, 7 and 11, respectively. In addition, positions of the remaining two long-tubed species, *P. batangensis* and *P. flexuosa*, are unresolved. It is curious that around half of the sampled long-tubed species are restricted to clade 3. In particular, the species of the *P. siphonantha* lineage are characterized by their purple/red/pink corollas with twisted beak. This implies that there is an association between having twisted-beaked corollas and geographic isolation. Except for *P. siphonantha* and its varieties [77], which have a relatively wide range of distribution, the other five species are each endemic to a small region, i.e., *P. dolichantha* endemic to the Huize region of northeastern Yunnan; *P. sigmoidea* and *Pedicularis* sp. (11) endemic to the Dali-Lijiang region and Ninglang region of northwestern Yunnan, respectively; and *P. leptosiphon* and *P. variegata* endemic to the Muli region of southwestern Sichuan [9, 29]. Moreover, geographic vicariance is found for the varieties of *P. siphonantha*, with var. *siphonantha* being restricted to the middle and western Himalayan region, var. *stictochila* widely occurring in western Sichuan at altitudes above 4000 m, and two unknown samples, *Pedicularis* sp. (12) and sp. (13) mainly distributed in northwestern Yunnan at altitudes below 4300 m. These latter two taxa have previously been generally misidentified as *P. siphonantha* var. *delavayi* or *P. delavayi*. However, Fig. 1c clearly demonstrates that *P. delavayi* is phylogenetically not part of the *P. siphonantha* lineage. Instead, it appears as the sister species of *P. obliquigaleata* of series *Dissectae*. Therefore, geographic isolation is an important mechanism to understand the diversification of *Pedicularis* in the Himalaya-Hengduan Mountains.

### Systematic implications for unresolved species

Among eight unresolved species, *P. axillaris* tended to be close to clade 3, *P. batangensis* to clade 1 or 4, while the systematic positions of the remaining six species (*P. flexuosa*, *P. lachnoglossa*, *P. mollis*, *P. moupinensis*, *P. petelotii* and *P. salviiflora*) are isolated (Fig. 1). Conservatively, these eight unresolved species are not treated as independent clades in this study. All eight are endemic to the Himalaya-Hengduan Mountains. Comparing them with the large clades 1, 3, 5 and 7, we suggest that the MRCAs of these eight species might not have experienced rapid radiation in the Himalaya-Hengduan Mountains, or else their relatives may have become extinct during the recent radiation, or be not yet sampled. Five unresolved species (*P. batangensis*, *P. lachnoglossa*, *P. mollis*, *P. moupinensis*, and *P. salviiflora*) belong to monotypic series [33], suggesting that the recently derived relatives might be extinct, or recent speciation might not have happened. Excluding *P. axillaris* and *P. lachnoglossa* with short (stem) branch lengths, the long (stem) branch lengths of the remaining six opposite-/whorled-leaved species indicate that they may have been derived at an early stage of the rapid radiation of *Pedicularis*. Due to the backbone of the tree being weakly supported, molecular dating may misinterpret their evolutionary histories. To improve phylogenetic resolution of the backbone, more DNA markers or next generation sequencing data should be applied.

## Conclusions

The flowers of *Pedicularis* show a great diversity in the form of the galea, with an extensive variation in the length of the corolla tube. Some natural groups are strongly supported by current phylogenetic analyses. Both nrITS and plastid datasets are relatively informative in *Pedicularis*; however, the relationships of the backbone branches of the phylogenetic tree are poorly resolved. Short branch lengths for the stem of 13 well-supported clades, together with long branch lengths for some tip branches/nodes, indicate that the MRCAs of *Pedicularis* may have undergone rapid radiations early. There is little consensus between this phylogeny and the taxonomic hierarchy of Tsoong’s system. Only two informal groups (15.4 %) and 19 series (33.9 %) are found to be strictly monophyletic. Our results suggest that combinations of morphological and geographic characters associated with strongly supported clades may be useful in developing a comprehensive global phylogeny of *Pedicularis*.

Phyllotaxy is a key character for infrageneric classification in *Pedicularis*. Most opposite-/whorled-leaved species fall into clade 1, and all whorled-leaved species with and perfoliate leaf bases that form a cup-like structure around the stem at each node are monophyletic in clade 4. Our results indicate that alternate leaves are inferred to be a symplesiomorphic character in *Pedicularis*, with multiple transitions to opposite/whorled phyllotaxy. Alternate-leaved species show high diversity in plant habit and floral forms, with most long-tubed species. In contrast whorled-leaved species are concentrated in the Himalaya-Hengduan Mountains and have many toothless species. Long-tubed corollas appear to have been derived from short-tubed corollas several times. Short-tubed species can bear all three types of galea (beakless and toothless, beakless and toothed, and beaked), while all long-tubed species are beaked. Our results suggest that geographical barriers may have facilitated diversification of long tubular species in the Himalaya-Hengduan Mountains. In addition, reproductive advantages may boost radiations of beakless galeas in opposite-/whorled-leaved species at high altitudes in the Himalaya-Hengduan Mountains.

### Availability of supporting data

The phylogenetic datasets (including amino acid sequence) supporting the results of this article are available in TreeBase (http://purl.org/phylo/treebase/phylows/study/TB2:S17733).

## Additional files

Additional file 1: Table S1. DNA accession and voucher information for taxa included in phylogenetic analysis. Additional file 2: Figure S1. Topology shows the majority rule consensus of the Bayesian inference tree using nrITS sequences. Values above the branches indicate ML bootstrap support (BS) ≥50, and those below the branches indicate BI posterior probability (PP) ≥0.50. Additional file 3: Figure S2. Topology shows the majority rule consensus of the Bayesian inference tree using the concatenated plastid dataset. Labeling details as Additional file 2.
