# Supplementary figures and images for: Towards a comprehensive phylogeny of the large temperate genus Pedicularis (Orobanchaceae), with an emphasis on species from the Himalaya-Hengduan Mountains
Source: BMC Plant Biol. 2015 Jul 11;15:176. doi: 10.1186/s12870-015-0547-9 (PMC4498522; doi:10.1186/s12870-015-0547-9)

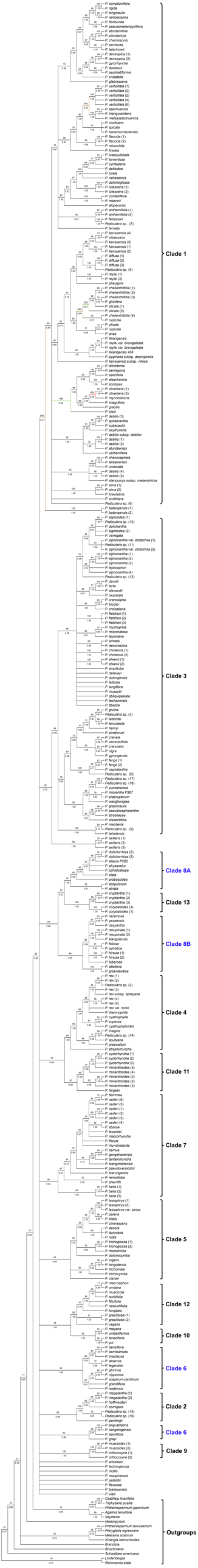

Supplement: Additional file 2: Figure S1. — Topology shows the majority rule consensus of the Bayesian inference tree using nrITS sequences. Values above the branches indicate ML bootstrap support (BS) ≥50, and those below the branches indicate BI posterior probability (PP) ≥0.50. [file 12870_2015_547_MOESM2_ESM.pdf]

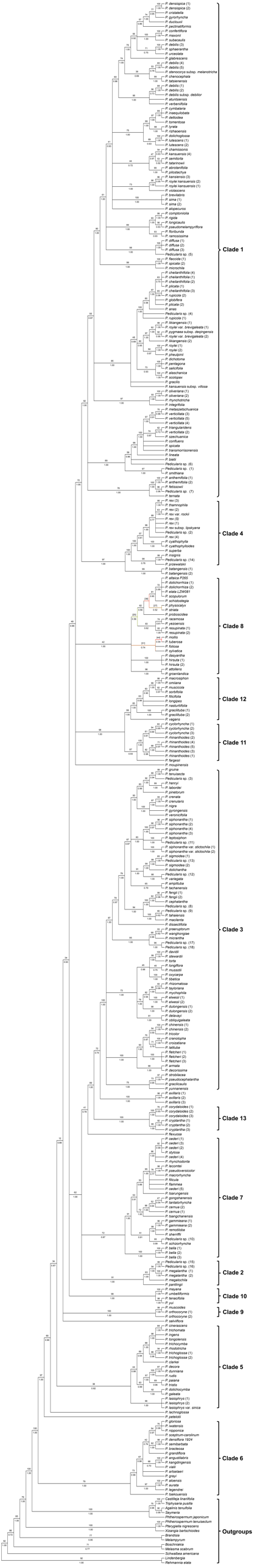

Supplement: Additional file 3: Figure S2. — Topology shows the majority rule consensus of the Bayesian inference tree using the concatenated plastid dataset. Labeling details as Additional file 2. [file 12870_2015_547_MOESM3_ESM.pdf]
